# Long-term effect of pregnancy-related factors on the development of endometrial neoplasia: A nationwide retrospective cohort study

**DOI:** 10.1371/journal.pone.0214600

**Published:** 2019-03-28

**Authors:** Hyun-Woong Cho, Yung-Taek Ouh, Kyu-Min Lee, Sung Won Han, Jae Kwan Lee, Geum Jun Cho, Jin Hwa Hong

**Affiliations:** 1 Department of Obstetrics and Gynecology, Guro Hospital, College of Medicine, Korea University, Seoul, Republic of Korea; 2 School of Industrial Management Engineering, Korea University, Seoul, Korea; Indiana University School of Medicine, UNITED STATES

## Abstract

**Objective:**

By identifying pregnancy-related risk factors for endometrial neoplasia, women’s risk of developing this disease after childbirth can be predicted and high-risk women can be screened for early detection.

**Methods:**

Study data from women who gave birth in Korea in 2007 were collected from the Korea National Health Insurance (KNHI) claims database between 2007 and 2015. The adjusted hazard ratios (HRs) and 95% confidence intervals (CIs) for the development of endometrial neoplasia were estimated by multivariate Cox proportional hazards models.

**Results:**

Data from 386,614 women were collected for this study. By 2015, 3,370 women from the initial cohort had been diagnosed with endometrial neoplasia secondary to delivery. Multivariate Cox proportional hazards regression revealed that preeclampsia (HR 1.55, 95% CI 1.29, 1.86), advanced maternal age (≥ 35; HR 1.52, 95% CI 1.39, 1.66), multifetal pregnancy (HR 1.81, 95% CI 1.46, 2.23), multiparity (HR 1.16, 95% CI 1.08, 1.24), cesarean section (HR 1.15, 95% CI 1.07, 1.23) and delivery of a large-for-gestational-age infant (HR 1.19, 95% CI 1.02, 1.39) were independent risk factors for future endometrial neoplasia. The risk for endometrial neoplasia increased as the number of risk factors increased (risk factors ≥3: HR 2.11, 95% CI 1.86–2.40).

**Conclusion:**

This study showed that six pregnancy-related factors—advanced maternal age, multiparity, multifetal pregnancy, cesarean section, delivery of a large-for-gestational-age infant, and preeclampsia—are positively correlated with future development of endometrial neoplasia, including endometrial hyperplasia or cancer. Close observation and surveillance are warranted to enable early diagnosis of endometrial diseases, including endometrial cancer after pregnancy in high-risk women. However, due to unavailability of clinical information, many clinical/epidemiological factors can become confounders. Further research is needed on factors associated with the risk of endometrial neoplasia.

## Introduction

Pregnancy can affect the endometrium in a variety of ways. During pregnancy, the size and number of glands and blood vessels in the endometrium increase significantly. The vascular space is fused and interconnected to form the placenta, which supplies oxygen and nutrients to the embryo and fetus [1]. Previous studies have shown that pregnancy reduces the risk of endometrial cancer by reducing estrogen exposure [2]; however, it has been reported that some pregnancy-related factors, such as placental growth factor (PlGF) and placenta-specific protein 1 (PLAC-1), are associated with endometrial cancer [3–7]. In addition, pregnancy-related Wnt signaling or Homeobox (HOX) genes are associated with endometrial cancer or endometrial disease [8–10].

Endometrial cancer is the most common cancer of the female genital tract worldwide [8, 11]. Risk factors for developing endometrial cancer include polycystic ovarian syndrome (PCOS), anovulatory infertility, obesity, age, family history and tamoxifen use, but there have been few studies of pregnancy-related risk factors for endometrial cancer [12]. Some epidemiologic studies have suggested that pregnancy reduces the incidence of endometrial cancer, and that the risk of endometrial cancer is further reduced by a greater number of pregnancies [13]. Although the results of previous studies examining associations between preeclampsia and endometrial cancer have been inconsistent [12, 14, 15], we hypothesized that pregnancy-related factors such as preeclampsia may be associated with endometrial neoplasia. More intensive risk-based screening of women after childbirth might lead to earlier detection pregnancy-related endometrial neoplasia.

## Materials & methods

### Healthcare system in Korea

Since 2000, the various health insurance systems in South Korea have been merged into a single system run by the National Health Insurance Service (NHIS). Consequently, most people living in South Korea are currently insured by the NHIS. Our study data were collected from the Korea National Health Insurance (KNHI) claims database from 2007–2015. In Korea, 97% of the population is obligated to enroll in the KNHI program; the remaining 3% are under the Medical Aid program. Therefore, the KNHI claims database contains information on all claims for approximately 50 million Koreans; nearly all information about disease incidence can be obtained from this centralized database, with the exception of procedures not covered by insurance, such as cosmetic surgery.

### Study population

**Fig 1:**
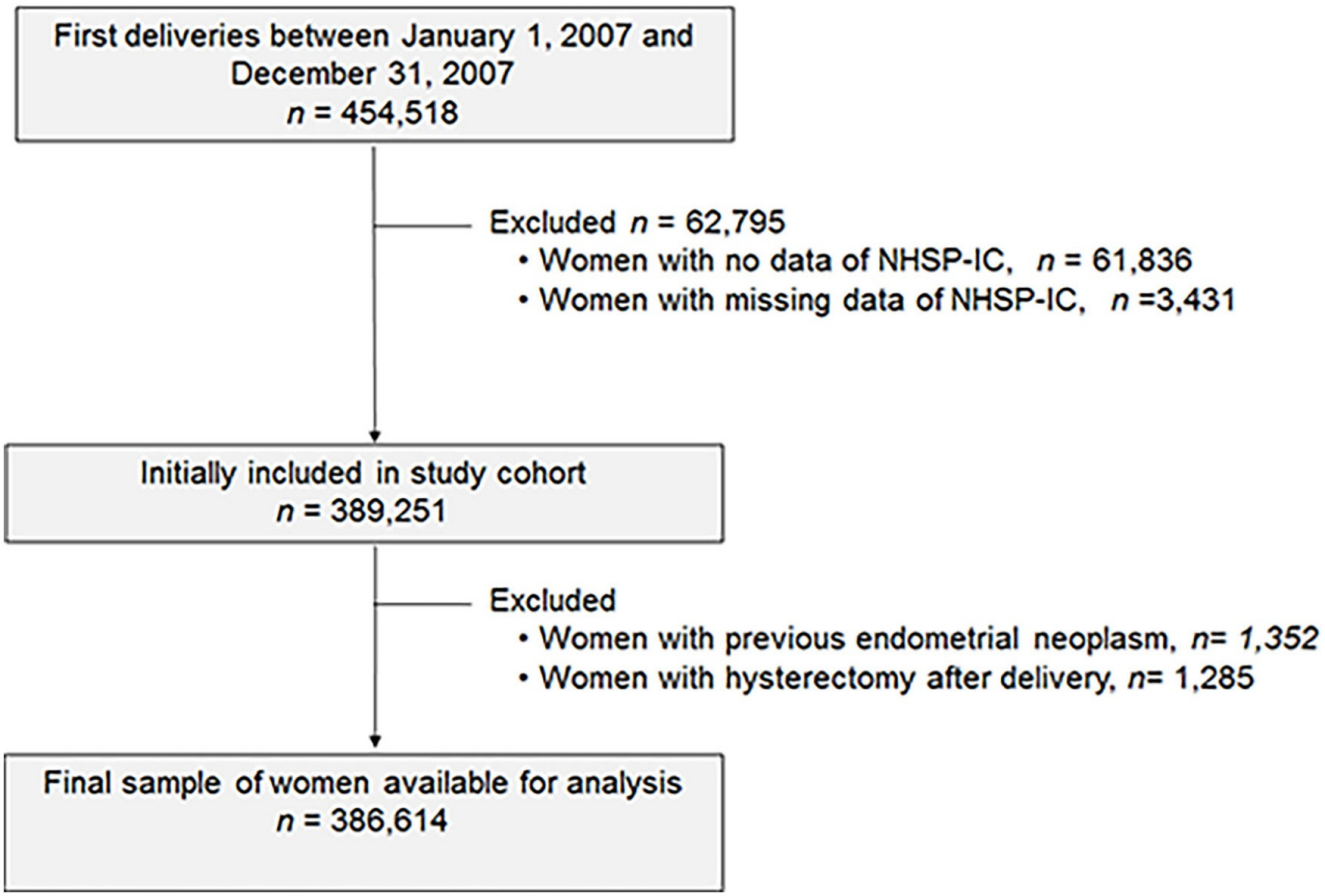
Flowchart of the retrospective cohort study design.

A flowchart of patient enrollment is shown in Fig 1. Using the KNHI claims database, we identified all women who gave birth between January 1, 2007 and December 31, 2007. Inclusion criteria were as follows: women who gave birth in 2007, had undergone the National Health Screening Program for Infant and Children (NHSP-IC) visit to evaluate neonatal characteristics, had no endometrial neoplasm before delivery, and did not undergo hysterectomy after delivery. Women with no NHSP-IC data, missing NHSP-IC data, previous endometrial neoplasia and hysterectomy were excluded in this study. This study was approved by the Institutional Review Boards of Korea University Medical Center (KUGH17256).

### Outcomes

Women diagnosed with endometrial neoplasia after their delivery were identified according to principal or secondary diagnosis by searching for relevant ICD-10 codes. Women were classified as having endometrial neoplasia if they were newly diagnosed with endometrial neoplasia (ICD-10 codes: N85.1A hyperplasia of endometrium, atypical, D07.0 carcinoma in situ of endometrium, and C54.1 malignant neoplasm of endometrium: endometrial cancer) from delivery to December 31, 2015. The timing of each patient’s initial diagnosis was confirmed by the lack of a diagnosis for endometrial neoplasia before pregnancy.

### Assessment of pregnancy characteristics

Pregnancy characteristics such as maternal age, primiparity, multifetal pregnancy, C/S, preeclampsia, postpartum hemorrhage, placental abruption, placenta previa, and uterine arterial embolization as identified by ICD-10 codes were evaluated using the KNHI claims database. Information about neonatal outcomes, specifically preterm birth, neonatal birthweight and gender, were analyzed using data from NHSP-IC. Preterm birth was defined as gestational age <37 weeks. Low birth weight (LBW) was defined as birth weight <2,500 g, and LGA as birth weight >4,000 g.

### Statistical analysis

Continuous and categorical variables were expressed as mean ± SD and percentages, respectively. Clinical characteristics were compared using the *t*-test for continuous variables and the *χ*^2^ test for categorical variables. Cox proportional hazards models were used to estimate the adjusted hazard ratios (HRs) and 95% confidence intervals (CIs) for the development of endometrial neoplasia. All tests were two-tailed, and p-values <0.05 were considered statistically significant. Statistical analyses were performed using SPSS version 18 (IBM Corp., Armonk, NY, USA).

## Results

Data from 386,614 women who gave birth in Korea from January 1, 2007 to December 31, 2007 were collected in this study. By 2015, 3,370 of these women had been diagnosed with endometrial neoplasia. Table 1 shows the pregnancy characteristics of participants with and without endometrial neoplasia.

**Table 1:** Pregnancy characteristics of participants stratified by the development of endometrial neoplasia.

Variables	No endometrial neoplasia (n = 383,244)	Endometrial neoplasia (n = 3,370)	p-value
Age (years) at birth	29.99 ± 3.78	30.75± 4.05	<0.001
Advanced maternal age (≥ 35; %)	11.61	17.86	<0.001
Multiparity (%)	45.45	50.12	<0.01
Multifetal pregnancy (%)	1.36	3.12	<0.001
Cesarean section (%)	35.92	41.60	<0.001
Preterm birth (%)	2.86	4.69	0.001
Neonatal birth weight (kg)	3.22±0.47	3.20±0.50	0.013
Low birth weight (%)	4.00	6.32	<0.001
Large for gestational age (%)	4.32	5.10	<0.001
Neonatal gender female (%)	51.56	50.47	0.210
Preeclampsia (%)	2.02	3.68	<0.001
Postpartum hemorrhage (%)	5.07	5.91	0.029
Placental abruption (%)	0.33	0.56	0.022
Placenta previa (%)	0.82	1.10	0.075
Uterine artery embolization (%)	0.08	0.15	0.147

In women with endometrial neoplasia, advanced maternal age, multiparity, multifetal pregnancy, C/S, preterm birth, neonatal birth weight, LBW, LGA, preeclampsia, postpartum hemorrhage, and placental abruption were more common than in women without endometrial diseases. The median age at birth was 30.75 years in patients with endometrial neoplasia and 29.99 years in patients without endometrial neoplasia. The proportion of women who developed endometrial neoplasia was two-fold greater in women who had multifetal pregnancies than in women without multifetal pregnancies. The proportions of preterm birth, low birth weight, and preeclampsia were more than 1.5 times greater in women who developed endometrial tumors after childbirth. However, no statistically significant difference was found between the two groups with respect to neonatal sex, placenta previa, and uterine artery embolization.

Table 2 shows the results of Cox proportional hazards regression analyses with significant variables. Advanced maternal age, multiparity, multifetal pregnancy, C/S, LGA, and preeclampsia were found to be independent risk factors for the development of endometrial neoplasia. Among the pregnancy-related variables, multifetal pregnancy (HR 1.81, 95% CI 1.46 to 2.23) was the strongest risk factor for endometrial neoplasia, followed by preeclampsia (HR 1.55, 95% CI 1.29 to 1.86) and advanced maternal age (HR 1.52, 95% CI 1.39 to 1.66).

**Table 2:** Multivariate Cox regression model of the risk of development of endometrial neoplasia.

Variables	Hazard ratios (HRs)	95% confidence intervals (CIs)
Advance maternal age (≥35)	1.52	1.39, 1.66
Multiparity	1.16	1.08, 1.24
Multifetal pregnancy	1.81	1.46, 2.23
Cesarean section	1.15	1.07, 1.23
Preterm birth	1.20	0.98, 1.47
Low birth weight	1.19	0.99, 1.42
Large for gestational age	1.19	1.02, 1.39
Neonatal gender female	0.95	0.89, 1.02
Preeclampsia	1.55	1.29, 1.86
Postpartum hemorrhage	1.15	0.99, 1.33
Placental abruption	1.42	0.90, 2.24
Placenta previa	1.11	0.80, 1.54
Uterine artery embolization	1.45	0.60, 3.52

Table 3 shows that the risk for developing endometrial neoplasia increases as the number of risk factors increases. Women with three risk factors were more than twice as likely to develop endometrial neoplasia as women without risk factors.

**Table 3:** Number of risk factors as a predictor of the development of endometrial neoplasia in Cox multivariate regression modeling.

Number of risk factors*	Hazard ratios (HRs)	95% confidence intervals (CIs)
0	1	
1	1.33	1.04, 1.24
2	1.55	1.42, 1.70
≥3	2.11	1.86, 2.40

*Risk factors: Advance maternal age (≥35), multiparity, multifetal pregnancy, cesarean section, large-for-gestational-age infant, preeclampsia

## Discussion

This study showed that six pregnancy-related factors—advanced maternal age, multiparity, multifetal pregnancy, C/S, LGA, and preeclampsia—are positively correlated with future development of endometrial neoplasia, including endometrial hyperplasia or cancer.

The most interesting finding in this study is that a history of preeclampsia is an important risk factor for endometrial neoplasia. Several studies have reported that the risk of breast cancer is reduced in women who have had preeclampsia [16, 17]. However, there have been few studies of the association between preeclampsia and endometrial cancer, and the results were inconsistent. A cohort study based on Swedish cancer registry data reported that there was no association between preeclampsia and endometrial, cervical, ovarian or breast cancer [14]. In contrast, the Jerusalem perinatal study suggested that the risk of all cancers was increased, especially cancers of the stomach (HR 6.45, 95% CI 2.16, 19.3), breast (HR 1.75, 95% CI 1.19, 2.58), and ovary (HR 3.25, 95% CI 1.15, 9.19), after preeclampsia [15]. Recently, it has been reported that preeclampsia was not associated with the development of endometrial cancer, but early-onset preeclampsia increased the likelihood of endometrial cancer, which was consistent with our study [12].

The inconsistent associations between preeclampsia and endometrial neoplasia reported in previous studies may be attributed to the different risk factors of two disease entities. The risk factors are different depending on ethnicity or race, suggesting that a history of preeclampsia might enhance the incidence of endometrial neoplasia in certain populations [18]. Genetic or lifestyle factors among different populations may also affect these outcomes. In addition, information regarding obesity and physical activity, which are important confounders, was not available in this study. Given obesity is an important risk factor for endometrial cancer and is a predisposing factor for preeclampsia [19, 20], obesity may have predisposed some mothers to develop preeclampsia and to also go on to develop endometrial neoplasia in this study.

The pathophysiology of preeclampsia has not been clearly elucidated, but preeclampsia is thought to begin in the placenta and may potentially have long-term impact on the endometrium. Preeclampsia may arise due to an imbalance of angiogenic and antiangiogenic factors (such as soluble fms-like tyrosine kinase Flt-1 [sFlt-1] and PlGF, an important pathogenic factor in the development of preeclampsia), which may affect endometrial vascular development or function during pregnancy. Most previous studies have focused on the antiangiogenic properties of sFlt-1 to treat cancer, while sFlt-1 was detected in colorectal and breast cancer tissue in some studies [21, 22]. Given sFlt-1 is frequently co-expressed with vascular endothelial growth factor (VEGF) in breast cancer, the balance between sFlt-1 and VEGF or PlGF has a significant impact on prognosis [23]. Most studies have reported that angiogenic factors such as VEGF and PlGF are associated with carcinogenesis. Conversely, preeclampsia which is associated with decreased VEGF or PlGF levels, was associated with an increased risk of endometrial neoplasia in this study. Hypothetically, hormone imbalances associated with angiogenesis due to preeclampsia may affect the endometrium rather than the level of each hormone, but the effects of these hormonal imbalances on the endometrium, especially the long-term effects, are not yet known. At present, it is difficult to explain the increase in endometrial neoplasia after preeclampsia based on the pathophysiology of preeclampsia, and additional research is needed to explore the relationship between these diseases.

In this study, advanced maternal age at birth was a risk factor for endometrial neoplasia. Many previous studies have shown that the risk of endometrial cancer decreases with increasing maternal age at delivery, while some studies have shown that the risk of endometrial cancer increases with increasing maternal age at delivery [24]. The conflicting results between studies may be attributed to confounders, such as contraceptive use and obesity [14, 25–28]

In this study, the incidence of endometrial neoplasia was found to increase in women who had multifetal pregnancies. This finding is similar to the results of a Swedish cohort study in which multiple births were associated with an increased risk of endometrial cancer (relative risk [RR] 2.32; 95% CI 1.02–5.25) [14]. The mechanism by which the history of multifetal pregnancies affects the development of endometrial neoplasia is unclear; however, there are several possible explanations. First, it has been reported that increased plasma concentrations of hormones such as GnRH and PlGF (secreted by the placenta), and proteins such as IGFBP1 and PLAC-1 are correlated to the development of endometrial cancer [29]. As the placenta is 1.9-fold heavier in twin versus single-fetus pregnancies [30], multifetal pregnancies may be expected to have a greater impact on the endometrium than singleton pregnancies. Second, infertility and ovulation induction resulting in multifetal pregnancies may also elevate the risk of endometrial cancer [31]. Many studies have reported inconsistent results regarding the association between infertility medication and endometrial cancer, but infertility drugs that elevate estradiol may cause hormone-related cancers, such as breast and endometrial cancer [32–35]. In addition to infertility drugs, infertility itself has been reported to be a risk factor for breast, ovarian and endometrial cancer [31]. However, one of the limitations of this study was that it was difficult to determine the rate of multifetal pregnancies after ART and the usage of infertility medications.

In this study, C/S was one of the risk factors for endometrial neoplasia. The association of C/S with endometrial cancer has rarely been studied. However, neo-angiogenesis with VEGF expression occurs within the cesarean section scar site of the uterus, which is thought to act as an endometrial cancer implant [36]. Other studies also have suggested that malignant transformation of endometriosis loci in C/S scars is possible [37–39]. As a result, the damage to the endometrium caused by C/S may lead to endometrial neoplasia. However, several studies have suggested that obesity may be a confounding factor. According to a meta-analysis, low socio-economic status (SES), high body mass index, gestational diabetes mellitus, and low maternal health status were reported as risk factors for cesarean section [40]. Considering the higher tendency to perform cesarean section in obese women, obesity appears to increase endometrial neoplasia in women who have undergone C/S.

Endometrial neoplasia was also increased in multiparous women in this study. These results are in contrast to those of many prior studies which reported having given birth to be a protective factor for endometrial cancer, and that the risk of endometrial cancer steadily decreases with increasing parity [2, 13, 41]. However, in a Swedish cohort study, the risk was greater for women who were parity 2 (RR 2.07) than in those who were parity 1 (RR 1.14) at 20–24 years of age and increased in parity 3–4 (RR 6.38) over parity 1 (RR 5.02) women aged 30 or older. In a cohort of black American women, the risk of endometrial cancer was reduced in parous women, but there was little evidence of a relationship between number of births and endometrial cancer risk [41].

It is possible that confounders such as obesity, oral contraceptive use, low SES and low maternal health status affected the outcomes in our study. Previous studies found positive associations between parity and obesity (38–40). In addition, it has been suggested that nulliparous women are more likely to take oral contraceptives that may have protective effects against endometrial cancer, and the tendency toward low SES, physical activity, and low maternal health status in obese patients may have affected the outcomes reported in some previous studies [42–46].

Similar to multifetal pregnancies, LGA also increased the risk of developing endometrial neoplasia. Placental weight is known to be lower with underweight babies and higher with overweight and LGA babies [47, 48]. Placentation process may have a greater effect on the endometrium in LGA and multifetal pregnancies. On the other hand, it is possible that LGA was not a risk factor, but rather that the characteristics of the mother acted as a risk factor. Obese mothers are at 1.5- to 2.5-fold greater risk of LGA development, and obesity is a well-known risk factor for endometrial cancer [31, 47]. Therefore, obesity may have confounded the relationship between LGA and endometrial neoplasia.

In this study, six risk factors were associated with endometrial neoplasia, and the greater the number of risk factors reported, the greater the risk. Patients with three or more risk factors were twice as likely to have an endometrial neoplasia. Although the risk factors identified in this study cannot be determined to have been the cause of the endometrial neoplasia reported in our study population, including endometrial hyperplasia and cancer, there was an increased risk of endometrial neoplasia in the women with these factors. The results of this study suggest evaluation of the risk of endometrial neoplasia immediately after delivery and close follow-up of high-risk patients may be helpful in early detection of endometrial cancer. In particular, women with three or more risk factors are at greater risk of developing endometrial neoplasia. Therefore, in such women, annual transvaginal ultrasonography and endometrial biopsy of suspicious lesions may be helpful in the prevention of endometrial cancer. In addition, efforts to reduce modifiable risk factors, such as C/S rate and multifetal pregnancies, through the use of ART may reduce the incidence of subsequent endometrial cancers. However, additional studies are needed to determine whether close follow-up and reduction of modifiable risk factors can effectively prevent endometrial neoplasia.

The study has several limitations. First, although six pregnancy related factors have been shown to increase the risk of endometrial neoplasia, none of them (advanced maternal age [HR 0.85, 95% CI 0.40 to 1.81], multi-parity [HR 1.90, 95% CI 0.43 to 8.51], multifetal pregnancy [HR 0.99, 95% CI 0.56 to 8.51], cesarean section [HR 1.49, 95% CI 0.82 to 2.70], delivery of a large-for-gestational-age infant [HR 0.51, 95% CI 0.12 to 2.21] and preeclampsia [HR 0.29, 95% CI 0.03 to 2.93]) have a statistically significant association with the risk of endometrial cancer. In this cohort, the sample size of cancer patients may not have been sufficient to show statistical significance, because the number of cancer patients among the 3,340 patients with endometrial neoplasia was only 75 (2.5%). Although six pregnancy-related factors do not increase the risk of cancer, atypical hyperplasia or endometrial carcinoma in situ can be considered precancerous lesions with a high malignancy potential. The 2014 World Health Organization (WHO) classified endometrial hyperplasia into two categories; hyperplasia without atypia, and atypical hyperplasia/endometrial intraepithelial neoplasia [49]. Although there is still controversy, most hyperplasia without atypia spontaneously resolves because there is no significant genetic change. In contrast, with atypical hyperplasia it is highly likely that endometrial cancer will coexist or develop into endometrial cancer within a few years, because it has many genetic mutations including microsatellite instability, PAX2 inactivation, phosphatase and tensin homolog (PTEN), KRAS, and CTNNB1 (β-catenin) [50–55]. Additional studies on the effects of the six pregnancy related factors investigated in this study on endometrial cancer will be needed.

Second, the study can be criticized for lack of clinical information in the HIRA database. Potential confounding variables including obesity, diabetes, hypertension, medication, and SES could not be controlled for due to unavailability of clinical information. However, the risk of cancer increased as the number of risk factors increased, although potential confounding variables might have affected the outcome.

Nonetheless, this study had several advantages. First, it is a large-scale study with a 10-year follow-up period. Second, it is the first study to examine the relationships between pregnancy-related risk factors and endometrial neoplasia. Finally, there was no loss to follow-up due to the nature of the data.

In this study, we confirmed the relationships between six pregnancy-related factors—advanced maternal age, multi-parity, multifetal pregnancy, cesarean section, delivery of a large-for-gestational-age infant and preeclampsia—and the occurrence of endometrial neoplasia (including endometrial hyperplasia and cancer) after pregnancy. Our results indicate close observation and surveillance may assist with the prevention and early diagnosis of endometrial neoplasia after pregnancy in high-risk women. However, there was no information on the many clinical factors such as BMI that could affect the outcome, and the pregnancy-related factors associated with endometrial cancer risk were not identified. Further studies on the relationship between pregnancy and future development of endometrial cancer will be needed.
