# Correction: Long-term effect of pregnancy-related factors on the development of endometrial neoplasia: A nationwide retrospective cohort study

**DOI:** 10.1371/journal.pone.0215979

**Published:** 2019-04-18

**Authors:** Hyun-Woong Cho, Yung-Taek Ouh, Kyu-Min Lee, Sung Won Han, Jae Kwan Lee, Geum Joon Cho, Jin Hwa Hong

The sixth author’s name is spelled incorrectly. The correct name is: Geum Joon Cho. The correct citation is: Cho H-W, Ouh Y-T, Lee K-M, Han SW, Lee JK, Cho GJ, et al. (2019) Long-term effect of pregnancy-related factors on the development of endometrial neoplasia: A nationwide retrospective cohort study. PLoS ONE 14(3): e0214600. https://doi.org/10.1371/journal.pone.0214600
